# Health-Related Quality of Life and Adherence to Hydroxyurea and Other Disease-Modifying Therapies among Individuals with Sickle Cell Disease: A Systematic Review

**DOI:** 10.1155/2022/2122056

**Published:** 2022-07-18

**Authors:** Mira Yang, Lena Elmuti, Sherif M. Badawy

**Affiliations:** ^1^Department of Medical Education, Northwestern University Feinberg School of Medicine, Chicago, IL, USA; ^2^Division of Hematology/Oncology, Comer Children's Hospital/University of Chicago, Chicago, IL, USA; ^3^Division of Hematology, Oncology, and Stem Cell Transplant, Ann & Robert H. Lurie Children's Hospital of Chicago, Chicago, IL, USA; ^4^Department of Pediatrics, Northwestern University Feinberg School of Medicine, Chicago, IL, USA

## Abstract

**Background:**

Sickle cell disease (SCD) is a hemoglobinopathy with increasing global prevalence resulting in pain episodes and multiorgan complications. Complications of SCD have been shown to adversely impact health-related quality of life (HRQOL) comprised of physical, social, and emotional domains; hence, HRQOL measures can serve as an effective evaluator of disease burden. Hydroxyurea (HU) and other disease-modifying therapies have demonstrated to significantly improve clinical outcomes in patients with SCD. Medication adherence is an essential mediator of the clinical benefits of these therapies; low adherence has been shown to increase disease burden and healthcare utilization. This systematic literature review intends to determine the association between adherence to disease-modifying therapies and HRQOL in patients with SCD.

**Methods:**

We found a total of 12 articles involving 788 participants, which included both patients with SCD and caregivers/parents. Adherence was measured using self-report instruments, laboratory markers, such as fetal hemoglobin and mean corpuscular volume, and mHealth medication trackers. HRQOL was measured using self-report instruments.

**Results:**

All studies demonstrated a correlation between higher HU adherence and better HRQOL scores. Higher HU adherence was associated with lower pain impact, less frequent pain episodes, less fatigue, and improved physical function and mobility, reflecting better physical HRQOL outcomes. Higher adherence was also associated with improved emotional response, decreased anxiety and depressive symptoms, and better social functioning and peer relationships. In addition, our findings indicated that having less frequent barriers to HU adherence was associated with better HRQOL scores. No studies evaluated HRQOL outcomes in relation to adherence to l-glutamine, voxelotor, or crizanlizumab.

**Conclusions:**

Optimizing HU adherence has the potential to improve HRQOL in patients with SCD in addition to reducing healthcare utilization and improving treatment satisfaction. Addressing barriers to HU adherence can positively strengthen the relationship between adherence and HRQOL to potentially improve patient outcomes.

## 1. Introduction

Sickle cell disease (SCD) is a hemoglobinopathy resulting from the inheritance of a point mutation in the beta chain, hemoglobin S, in the *β*-globin gene [1]. It is the most common monogenic disorder with an increasing global prevalence; there are an estimated 100,000 individuals in the U.S. alone with SCD [2–4]. Many different genetic variants of SCD exist, including homozygous HbSS variants and compound heterozygous forms such as HbSC, HbS-*β*^+^ thalassemia, and HbS-*β*^0^ thalassemia [2, 5, 6]. HbSS is the most severe variant of SCD due to increased levels of sickled hemoglobin, caused by red blood cells, with HbS undergoing polymerization, leading to increased rigidity and hemolysis in deoxygenated environments [1, 7]. These dense, rigid red blood cells lead to multisystem, multiorgan complications through mechanisms related to vasoocclusion, tissue ischemia, and infarction [8]. The two main groups of chronic complications include large-vessel vasculopathy, such as cerebrovascular disease and pulmonary hypertension, and progressive ischemic organ damage, such as hyposplenism and renal failure [2]. The most common acute complication of SCD is acute vasoocclusive episodes (VOE), also known as sickle cell pain episodes [1].

As a result of the systemic damage imparted by this chronic and often debilitating disorder, complications of SCD have been shown to adversely impact and impair health-related quality of life (HRQOL) within the physical, social, and emotional health domains [2, 9]. These domains are highly interconnected and can be triggered by external stressors and social determinants of health. Currently, the available disease-modifying therapies for SCD are hydroxyurea (HU), voxelotor, l-glutamine, and crizanlizumab [10, 11]. HU is the most utilized disease-modifying medication with a well-established clinical efficacy and has been helpful in decreasing the frequency of VOE episodes and acute chest syndrome events. Treatment with HU is also associated with decreased health care utilization, costs, and risk of early mortality [10, 12, 13]. Voxelotor binds to the high-oxygen affinity, nonpolymerizing conformation of HbS, leading to increases in hemoglobin concentration [10, 14]. L-Glutamine and crizanlizumab can be used concurrently with HU to reduce pain episodes or may be used if HU is not tolerated or is ineffective in patients with SCD [11, 15].

Medication adherence is essential to achieve the targeted outcomes of these disease-modifying therapies. Nevertheless, HU adherence is suboptimal among SCD patients [16–19]. Despite the myriad of reported benefits of HU in SCD, there are several reasons why patients choose not to adhere to HU, including fears about side effects, barriers to receiving refills or accessing medication, difficulty with daily recall, and lack of patient engagement and autonomy in decision-making [20–22]. More recently, methods of implementing HRQOL measures into practice have increased and gained momentum. Quantifying patients' symptoms as well as enhancing their autonomy and shared decision-making between physicians and patients are all potential benefits of routine use of HRQOL measures in clinical practice settings. The objective of this systematic review was to assess the relationship between HRQOL outcomes and adherence to various disease-modifying therapies among SCD patients.

## 2. Methods

This systematic review was completed according to the Preferred Reporting Items for Systematic Reviews and Meta-Analysis (PRISMA) guidelines [23]. Studies that were included in this study involved patients with any SCD genotype (i.e., HbSS, HbSC, HbS/*β*^0/+^ thalassemia, and other heterozygous variants of SCD), evaluated HRQOL outcomes and adherence to any disease modifying therapies using reliable and validated measures or instruments, and were cross-sectional or longitudinal studies with 5 or more participants. We only included studies that used reliable and validated measures to allow for more consistency in measured outcomes and comparisons of the studies. Case reports of less than 5 participants, reviews, viewpoints, editorials, letters to the editor, animal studies, and studies of laboratory investigations were excluded. Main outcomes included patient- and/or parent-reported HRQOL scores and self-report or laboratory markers of medication adherence.

PubMED, MEDLINE, and Cochrane Central Register of Controlled Trials (CENTRAL) on the Wiley platform were the databases used to complete the literature search for this review with no language restrictions. Articles from 1981 until 2021 were indexed without any restriction on the publication date. The search terms used were a combination of (1) sickle cell disease AND (2) health-related quality-of-life OR quality-of-life. Our search was further filtered to focus on studies involving medication adherence with an FDA-approved disease-modifying therapy for SCD. An additional search was conducted in January 2022 following the same criteria. We also scanned the reference lists of any included articles to look for other relevant studies.

## 3. Results

### 3.1. Literature Search

**Figure 1:**
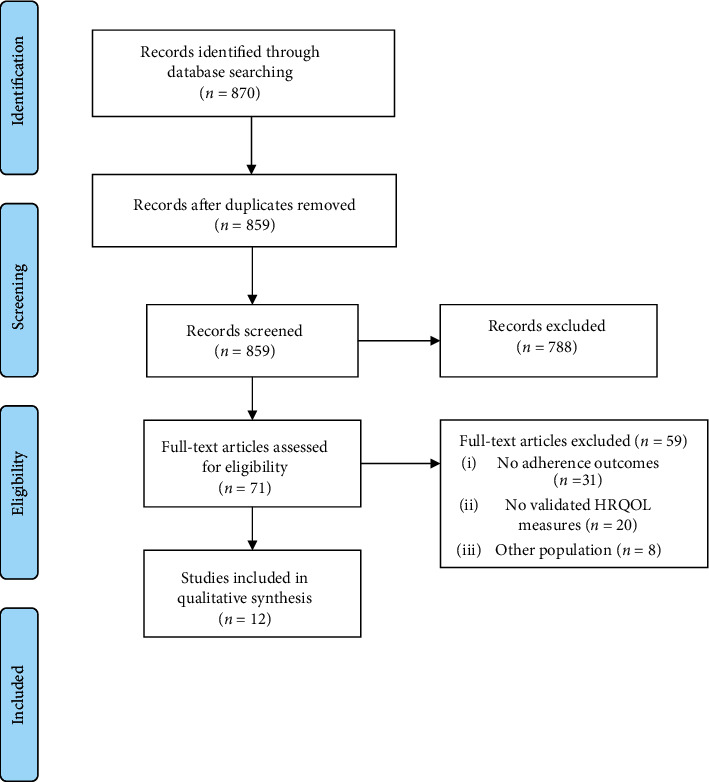
Flow of studies through the review according to the PRISMA guidelines.

The literature search identified 859 articles. Of these, 788 articles were excluded based on title and abstract screening, and 71 articles were retrieved for full-text screening. Twelve articles met all inclusion criteria and were included in this review (Figure 1).

### 3.2. Description of All Included Studies

All 12 studies assessed patients with SCD of multiple genotypes. A total of 788 participants, including patients with SCD and/or their parents or caregivers took part in the included studies and reported HRQOL outcomes. Studies collected data from patients only (*n* = 9, 75%) [21, 22, 24–30], both parent/proxy and self-report (*n* = 2, 16.7%) [31, 32], or caregivers only (*n* = 1, 8.3%) [33]. All twelve studies enrolled adolescent participants, while two (16.7%) enrolled adults (age > 18 years old) [28, 30]. All included studies used at least one validated HRQOL measure, and 4 studies (33.3%) used multiple HRQOL instruments [28, 29, 31, 32].

### 3.3. Description of Study Characteristics in SCD

A summary of the characteristics of all studies included in this review are included in Table 1. Seven studies (58.3%) used the Patient-Reported Outcomes Measurement Information System (PROMIS) scale alone to evaluate HRQOL outcomes [21, 22, 24–27, 30]. Four of the studies (33.3%) used the Pediatric Quality of Life Inventory (PedsQL), Pediatric Quality of Life Sickle Cell Disease Module (PedsQL SCD), Pediatric Quality of Life Multidimensional Fatigue Scale (PedsQL MFS), and/or the parent-proxy of the PedsQL instrument(s) [29, 31–33], while one (8.3%) used Profile of Mood States (POMS) and the 36-Item Short Form Survey (SF-36 survey) [28]. Regarding measurement of medication adherence, six studies (50%) used the Morisky Medication Adherence Scale (MMAS-8) to measure HU adherence [21, 22, 24–26, 30], three (25%) used the Visual Analogue Scale (VAS) [22, 27, 30], and five (41.7%) used laboratory markers, such as fetal hemoglobin (HbF) and/or mean corpuscular volume (MCV) [21, 22, 25, 28, 32]. One study (8.3%) developed a mobile intervention (ITP app) [29], used the Adherence & Self-Care Inventory (ASCI) tool [33], or used the Parent Medication Barriers Scale (PMBS) and Adolescent Medication Barriers Scale (AMBS) to measure adherence [31]. Five studies (41.7%) used multiple measurements of adherence [21, 22, 24, 30, 31].

Nine studies (75%) were categorized as cross-sectional studies [21, 22, 24–27, 30, 31, 33], while the remaining three (25%) were categorized as longitudinal studies [28, 29, 32]. Patient ages ranged from 0 to 66, with the reported mean age ranging from 10 to 15 years and medians ranging from 11 to 17 years. The average number of SCD participants per study was 66 with a median of 34 participants (range 32-299). Eleven of the studies (91.7%) were conducted in the United States [21, 22, 25–29, 31–33], with only one conducted in the UK [30]. Four studies did not report the specific SCD genotype for included patients [26, 28, 31, 32]. Eight studies (66.7%) included known HbSS patients [21, 22, 24, 25, 27, 29, 30, 33], seven (58.3%) included HbSC patients [21, 22, 24, 25, 27, 29, 33], seven (58.3%) included HbS-*β*^0^ and HbS/*β*^+^ Thal patients [21, 22, 24, 25, 27, 29, 33], and one (8.3%) included Hb SO-Arab patients [29].

### 3.4. Association between Medication Adherence and QoL Outcomes

All studies synthesized for this review (*n* = 12, 100%) demonstrated a correlation between higher HU adherence and better HRQOL scores. This includes improvements in overall HRQOL scores as well as in individual components of wellbeing and hospitalization (Table 2). The significance and strength of the relationships varied across studies due in part to HRQOL metrics used, patient demographics, and adherence scales used, among other factors. Low adherence was measured as an MMAS-8 score from 0 to <6, a VAS score ≤ 80% and an ITP app entry rate ≤ 75% [21, 22, 24–27, 29, 33].

Improvements in pain impact were a focus of some analyses; higher adherence rates were associated with lower pain impact, better physical function of the upper extremities, and improved physical function mobility [26, 27, 29]. Pain episode frequency was strongly correlated with both adherence and HRQOL scores; patients with higher adherence and better HRQOL scores had fewer pain episodes [33]. In addition to pain, fatigue was one of the most common measures of quality of life noted to be lower in patients with lower adherence to HU [24]. Higher adherence was inversely correlated with fatigue; similarly, some patients reported reduced tension when adherence to HU was higher [21, 24, 28].

Physical complications of SCD were supported by biomarker measures including HbF and MCV. Using a fetal hemoglobin (HbF) cutoff of 10%, participants with low HbF% scores reported worse SCD-related emotional response and less perceived benefits of HU [21, 22, 24]. Intervention group participants who received tailored text messages designed to improve HU adherence reported increased personal best HbF by 2.3% during months 0-4, with three intervention group subjects exceeding their historical personal best HbF by study completion [32]. Participants assigned to HU with high two-year response to HbF had better “general health now” scores, pain recall, and general health perception scores compared to those with low two-year HbF response [28]. Patients with high MCV, which was positively correlated with adherence scores, reported better emotional response and more perceived benefits of HU compared to those with low values [21, 24]. Furthermore, patients with lower MCV values (<102 fl) reported worse fatigue, pain, physical function, mobility, depression, and social isolation scores compared with those with high MCV values [24].

Different facets of mental health, including depression and anxiety, were also analyzed in several of our included studies. Patients with lower adherence scores tended to report worse depression and/or anxiety [21, 24, 27]. Social isolation, peer relationship, and social functioning scores were also worse in patients with lower adherence scores [21, 24, 27, 28]. One study focusing on the effects of adherence to HU on hospitalization noted that participants with greater adherence to HU had shorter hospital stays, and patients with no hospitalization reported significantly higher median adherence scores [26]. Another study found that compared to those with 1-3 hospitalizations, participants with 4 or more hospitalizations perceived increased SCD-related symptoms and consequences, resulting in a worse emotional response to SCD [21]. Studies focusing on the relationship between barriers to HU adherence and HRQOL supported these findings, as increased pain crisis frequency was one of the largest contributors to HRQOL [33]. Having barriers to treatment adherence in general affected HRQOL scores, a greater number of total barriers to adherence was inversely associated with total generic and disease specific HRQOL scores [31, 33]. Commonly reported barriers include forgetfulness, knowledge deficits, and access barriers such as issues with medication refills [22, 31]. Meanwhile, reducing barriers to adherence such as through the use of visits and text message reminders improved adherence and HRQOL scores [32].

## 4. Discussion

HU is a well-known disease-modifying drug; however, the impact of HU adherence on the HRQOL of patients with SCD is not clearly understood. Hence, the 12 studies analyzed in this review focused on the relationship between HU adherence and HRQOL. In addition, some studies focused on potential moderators of adherence such as barriers to adherence and healthcare utilization. Overall, patients with lower adherence tended to have worse HRQOL scores. Adherence to HU was inversely correlated to pain, fatigue, depression, anxiety, and tension; adherence was positively correlated to social functioning/wellbeing, emotional response, and perceived benefits of HU.

There are a variety of tools that can be used to monitor medication adherence in patients with SCD. Within the studies we analyzed, self-reported adherence surveys were the mostly commonly used tool, such as the MMAS-8 and VAS, followed by laboratory markers such as MCV and HbF levels. One study employed a new mHealth medication tracking tool to measure adherence [29]. Other research studies have used pharmacy dispensing data, medication discontinuation/continuation rates, public insurance claims, and/or pill counting to measure adherence [34, 35]. Clinically, poor adherence to HU has been shown to adversely affect laboratory evidence resulting in lower MCV values and HbF% [24, 36]. Deviation from historical “personal best” HbF levels has been shown to be associated with changes in adherence levels [36]. Increased deviation from “personal best” HbF levels was associated with lower MCV, and MCV has also been positively correlated with MMAS-8 self-report adherence scores [21, 36]. Use of a medication tracking app supplemented by medical record review yielded positive correlations between adherence and HRQOL scores, as did usage of visits and medication reminders, which corresponded in part with higher adherence [29, 32]. Preliminary research has supported mHealth as tools to potentially increase medication adherence in SCD patients; these tools have the potential to increase education on maintenance medications, provide refill and visit reminders, and track medication usage [19, 37]. Some trials have already demonstrated some benefit of mHealth tools in raising adherence rates, although these tools must undergo continued research before widespread dissemination [32, 38].

Increased barriers to HU adherence were more likely to worsen HRQOL as well [22, 31, 33]. mHealth tools, as outlined above, may help to combat one of the most common barriers of forgetfulness [22, 31, 32]. Another commonly cited reason for poor treatment adherence in patients with SCD were patient concern and perception of HU. Lower perceived benefit of HU was associated with more frequent hospitalization and emergency department (ED) visits [21, 26]. Patients with increased fear towards HU had lower adherence rates and worse quality-of-life scores [21, 27]. Delving into individual patient or parent misconceptions or fears regarding SCD and HU through counseling may increase provider patient agreement and awareness [39, 40]. Inclusion of barriers to adherence to the regression model of adherence and HRQOL led to a nonsignificant association, emphasizing the need to address barriers to adherence in addition to studying adherence itself [33]. Research specifically focusing on barriers to HU adherence has reported access to HU, fear of side effects and efficacy, and decreased education as being linked to worse symptoms of pain, fatigue, and depression [22]. Barriers to adherence can contribute to a negative feedback loop of decreased belief in medication, moderating decreased adherence. Overall, identifying barriers as part of routine patient care could help with patient adherence to HU to improve HRQOL.

Our systematic review has several strengths. First, we conducted this review following the PRISMA methodology for systematic reviews [23]. To minimize publication bias, a search strategy was devised to identify as many relevant research studies as possible. Articles since 1981 were indexed even though the first eligible article was from 2003. Therefore, the possibility of missing studies published earlier is small. In addition, two authors completed the review process independently at all stages. There are, however, potential limitations to our systematic review of the literature. As with any systematic review, there is a possibility of missing relevant articles despite developing a comprehensive search strategy. As only articles published in peer-reviewed journals were included, there is a possibility of publication bias. Varying sample sizes, ages, and measurement tools prevented a meta-analysis from being performed. In particular, it is worth noting that barriers to HU adherence are likely different across age groups, such as adolescents compared to young children or adults. Adolescents have unique developmental and behavioral changes as they navigate their increased responsibilities balancing school, work, and social activities as well as transition to adult care. These challenges are more profound in SCD where patients are also at increased risk of cognitive and executive function impairment along with limited adaptive functioning and self-care independence. Finally, many studies included sample sizes that were relatively small, and few articles were related to the same study that was conducted at a single large academic medical center with a modest sample size.

## 5. Conclusion

In this review, we evaluated the impact of adherence to HU on HRQOL. In general, HU as a treatment not only improves health outcomes clinically for patients with SCD by decreasing disease complications and reducing mortality; it also has a potential impact on mental and social wellbeing. Our review identified the potential for improved HU adherence to improve HRQOL as well as reduce healthcare utilization, pain episodes, and dissatisfaction with medication efficacy and side-effect profile. Addressing barriers to HU adherence can additionally positively strengthen the relationship between adherence and HRQOL.

Ongoing inclusion of patient-reported HRQOL screening in the inpatient and outpatient setting continues to be vital in improving HU adherence and thereby improving quality-of-life. Understanding the impact of this drug on comprehensive quality of life, including factors such as patient perception of the drug and barriers to treatment, is vital in improving medical adherence to HU and working to improve overall long-term prognosis in patients who suffer from SCD.

## Figures and Tables

**Table 1:** Summary study characteristics and data collection tools.

Author, year	Country, # of participants	Patient age	Sickle cell genotype	Study type	QOL tool	Adherence tool
Anderson et al., 2018 [29]	USA Patients: 32	Mean: 13.0 Range: 7-18	HbSS, HbSC, sickle-*β*0 Thal, S-O Arab	Longitudinal study	PedsQL, PedsQL-SCD, Peds-QL MFS	ITP app and medical record review
Badawy et al., 2016 [24]	USA Patients: 34	Median: 13.5 Range: 12-18	HbSS, HbSC, HbSB	Cross-sectional study	PROMIS	MMAS-8, MCV, HbF biomarker
Badawy et al., 2017 [21]	USA Patients: 34	Mean: 14.8 Median: 13.5 Range: 12-18	HbSS, HbSC, HbSB	Cross-sectional study	PROMIS	MMAS-8, MCV, HbF biomarker
Badawy et al., 2017 [22]	USA Patients: 34	Median: 13.5 Range: 12-18	HbSS, HbSC, HbSB	Cross-sectional study	PROMIS	MMAS-8 VAS, MCV, HbF biomarker
Badawy et al., 2018 [25]	USA Patients: 34	Mean: 14.8 Median: 13.5 Range: 12-18	HbSS, HbSC, HbSB	Cross-sectional study	PROMIS	MMAS-8
Badawy et al., 2018 [27]	USA Patients: 34	Median: 13.5 Range: 12-18	HbSS, HbSC, HbSB	Cross-sectional study	PROMIS	VAS
Badawy et al., 2019 [26]	USA Patients: 34	Median: 13.5 Range: 12-18	Did not report	Cross-sectional study	PROMIS	MMAS-8
Ballas et al., 2006 [28]	USA Patients: 299	Median: not reported Range: 0-66	Did not report	Longitudinal study	SF-36 POMS	HbF biomarker
Fisak et al., 2010 [33]	USA Caregivers: 78	Median: 11 Range: 5-17	HbSS, HbSC, sickle-*β*+ Thal, sickle-*β*0 Thal	Cross-sectional study	Parent proxy of PedsQL 4.0	ASCI
Fogarty et al. 2021 [30]	UK Patients: 63	Median: 17 Range: 12-35	HbSS	Cross-sectional study	PROMIS (adapted)	VAS Adapted: MMAS-8
Smaldone et al., 2018 [32]	USA Subjects: 56 (28 youth-parent dyads)	Mean: 14.3 Range: not reported	Did not report	Longitudinal study	PedsQL and PedsQL SCD	HbF biomarker
Smaldone et al., 2019 [31]	USA Subjects: 56 (28 youth-parent dyads)	Range: 10-18	Did not report	Cross-sectional study	PedsQL and PedsQL SCD	PMBS, AMBS

Abbreviations: AMBS: Adolescent Medication Barriers Scale; ASCI: Adherence & Self-Care Inventory; HbF: fetal hemoglobin; MCV: mean corpuscular volume; MMAS-8: Morisky Medication Adherence Scale: PedsQL: Pediatric Quality of Life Inventory; PedsQL MFS: Pediatric Quality of Life Multidimensional Fatigue Scale; PedsQL SCD: Pediatric Quality of Life Sickle Cell Disease Module; PMBS: Parent Medication Barriers Scale; POMS: Profile of Mood States; SF-36: 36-Item Short Form Survey; VAS: Visual Analogue Scale.

**Table 2:** Summary of study findings evaluating adherence to HU and HRQOL.

Author, year	Main results
Anderson et al., 2018	(i) Participants with daily ITP app entry rate ≥ 0.75 (completers) reported better SCD-related functioning and parent-reported treatment functioning as well as lower pain impact (*p* < 0.05) (ii) Participants with daily ITP < 0.75 (noncompleters) demonstrated worsening of pain impact scores (*p* < 0.05) (iii) Completers reported poststudy pain impact scores near or above clinical cutoff for good clinical functioning (65.8 child-reported scores vs. 27.5 for noncompleters)

Badawy et al., 2016	(i) Participant adherence scores were correlated with fatigue (*p* = 0.01) and social isolation scores (*p* =0.02) (ii) MMAS-8 adherence scores were positively correlated with HbF (*p* = 0.04), and participants with low MMAS-8 scores had significantly lower MCV values (*p* = 0.001); participants with lower HbF% scores had worse social isolation and fatigue scores (iii) Participants with low MCV values reported worse fatigue, pain, physical function mobility, depression, and social isolation scores

Badawy et al., 2017^a^	(i) Patients with better adherence to HU perceived more benefits from HU (*p* < 0.01) and had a better emotional response to SCD (*p* = 0.01) (ii) MMAS-8 scores positively correlated with fetal hemoglobin (HbF) and mean corpuscular volume (MCV) and was inversely correlated with fatigue, depression, social isolation (iii) Patients with more negative perceptions of their disease and less perceived benefits of HU reported worse fatigue (*p* = 0.03), anxiety (*p* < 0.01), and depression (*p* < 0.001)

Badawy et al., 2017^b^	(i) Barriers like access to HU, fear about drug side effects and efficacy, and decreased education on HU were mentioned linked to worse pain, fatigue, and depression (ii) Patients who were fewer barriers had better adherence to HU and improved quality of life scores (iii) The number of adherence barriers was inversely correlated with MCV values (*p* = 0.01) and HbF% (*p* = 0.05)

Badawy et al., 2018^a^	(i) Female patients and patients who were older had lower quality of life scores (ii) 74% of participants in this study had poor adherence to HU (iii) Male patients were noted to have better quality of life scores and better adherence than females (iv) There were no significant differences in self-reported adherence to HU among patients of different age groups noted in this study

Badawy et al., 2018^b^	(i) Participants with high HU adherence (VAS ≥ 80%) had significantly fewer concerns about HU (*p* = 0.02); participants' concerns positively correlated with anxiety (*p* = 0.01) and depression (*p* = 0.001) and were inversely correlated with peer relationships (*p* = 0.03) and physical functioning of upper extremities (*p* = 0.05)

Badawy et al., 2019	(i) Participants with greater adherence to HU had shorter hospital stays (*p* = 0.06) (ii) Participants with no hospitalizations reported significantly higher median adherence scores (*p* = 0.03) (iii) Participants with 1+ hospitalizations reported worse median scores for fatigue (*p* = 0.02), pain (*p* = 0.03), and physical function mobility (*p* = 0.001)

Ballas et al., 2006	(i) Benefits of HU treatment adherence included benefits in present general health (*p* < 0.001), pain recall (*p* = 0.004), social functioning (*p* = 0.007), and general health perception (*p* = 0.001) (ii) Some patients also reported a reduction in tension when adherent to HU (*p* = 0.001)

Fisak et al., 2010	(i) Barriers to treatment adherence and increased pain crisis frequency were the largest contributors to health-related quality of life (ii) Adherence was associated with HRQOL (*p* < 0.01); inclusion of barriers to adherence to the regression model led to nonsignificant association between adherence and HRQOL

Fogarty et al., 2021	(i) Participants with >80% HU adherence perceived more beneficial effects of medication compared to those with ≤80% adherence (*p* = 0.06)

Smaldone et al., 2018	(i) Patients receiving visits and text message reminders to improve adherence reported improved generic and disease-specific HRQOL scores in all categories

Smaldone et al., 2019	(i) A greater number of total barriers to adherence was inversely associated with total generic and disease specific HRQOL scores (*p* < 0.001 and *p* < 0.001 for youth-reported scores)

## Data Availability

The data generated from this study are available upon request. All data in this systematic review were generated from the included published studies.
